# Multidrug-Resistant *Staphylococcus aureus* Colonizing Pigs and Farm Workers in Rio de Janeiro State, Brazil: Potential Interspecies Transmission of Livestock-Associated MRSA (LA-MRSA) ST398

**DOI:** 10.3390/antibiotics13080767

**Published:** 2024-08-14

**Authors:** Joana Talim, Ianick Martins, Cassio Messias, Hellen Sabino, Laura Oliveira, Tatiana Pinto, Julia Albuquerque, Aloysio Cerqueira, Ítalo Dolores, Beatriz Moreira, Renato Silveira, Felipe Neves, Renata Rabello

**Affiliations:** 1 Department of Microbiology and Parasitology, Instituto Biomédico, Universidade Federal Fluminense, Niterói 24020-150, RJ, Brazil; jotavares@id.uff.br (J.T.); hellensabino@id.uff.br (H.S.); jp_albuquerque@id.uff.br (J.A.); aloysiocerqueira@id.uff.br (A.C.); fpgneves@id.uff.br (F.N.); 2 Faculty of Medicine, Universidade Federal Fluminense, Niterói 24033-900, RJ, Brazil; ianicksm@id.uff.br; 3 Centro de Ciências Biológicas e da Natureza, Universidade Federal do Acre, Rio Branco 69915-900, AC, Brazil; cassio.messias@ufac.br; 4 Instituto de Microbiologia Paulo de Góes, Universidade Federal do Rio de Janeiro, Rio de Janeiro 21941-902, RJ, Brazil; lauraoliveira@micro.ufrj.br (L.O.); tcap@micro.ufrj.br (T.P.); bmeurer@micro.ufrj.br (B.M.); 5 Departament of Clinical Medicine, Faculty of Medicine, Universidade de São Paulo, São Paulo 01246-903, SP, Brazil; italodolores@usp.br; 6 Department of Morphology, Instituto Biomédico, Universidade Federal Fluminense, Niterói 24210-130, RJ, Brazil; renatosilveira@id.uff.br

**Keywords:** *Staphylococcus aureus*, LA-MRSA, multidrug resistance, pig, livestock, farm worker

## Abstract

Multidrug-resistant (MDR) *Staphylococcus aureus* has been increasingly isolated from pigs and people in close contact with them, especially livestock-associated methicillin-resistant *S. aureus* (LA-MRSA). In this cross-sectional study, we investigated *S. aureus* colonization in pigs and farm workers, their resistance profile, and genetic background to estimate interspecies transmission potential within farms from Rio de Janeiro state, Brazil, between 2014 and 2019. We collected nasal swabs from 230 pigs and 27 workers from 16 and 10 farms, respectively. Five MDR strains were subjected to whole genome sequencing. Fourteen (6.1%) pigs and seven (25.9%) humans were colonized with *S. aureus*, mostly (64–71%) MDR strains. Resistance to clindamycin, erythromycin, penicillin, and tetracycline was the most common among the pig and human strains investigated. MDR strains shared several resistance genes [*blaZ*, *dfr*G, *fexA*, *lsa*(E), and *tet*(M)]. Pig and human strains recovered from the same farm shared the same genetic background and antimicrobial resistance profile. LA-MRSA ST398-SCC*mec*V-t011 was isolated from pigs in two farms and from a farm worker in one of them, suggesting interspecies transmission. The association between pig management practices and MDR *S. aureus* colonization might be investigated in additional studies.

## 1. Introduction

Pork meat is a highly consumed protein source around the world, leading swine farms to seek practices and technologies that increase productivity. However, such practices often facilitate the spread of infectious diseases, increasing the use of antimicrobial agents. These drugs are widely used both to treat and prevent bacterial infections in many countries. In some countries, their use is still allowed as performance enhancers [1,2]. Over the years, the use of several classes of antimicrobial agents as performance enhancers has been banned in Brazil, such as amphenicols, tetracyclines, beta-lactams, quinolones, sulfonamides, macrolides, and lincosamides [https://www.gov.br/agricultura/pt-br/assuntos/insumos-agropecuarios/insumos-pecuarios/resistencia-aos-antimicrobianos/legislacao/proibicoes-de-aditivos-na-alimentacao-animal, accessed on 6 June 2024]. Recently, new legislation came into force that establishes new rules and procedures for the manufacture of products intended for animal feeding with medicines for veterinary use [https://www.gov.br/agricultura/pt-br/assuntos/insumos-agropecuarios/insumos-pecuarios/alimentacao-animal/PORTARIASDAN798DE10DEMAIODE2023PORTARIASDAN798DE10DEMAIODE2023DOUImprensaNacional.pdf, accessed on 6 June 2024]. 

Selective pressure due to antimicrobial use can lead to the selection of multidrug-resistant (MDR) bacteria in farms [3]. Infections with MDR bacteria are a global public health concern [4]. Currently, control of antimicrobial resistance emergence relies on the One Health approach, covering human, animal, and environmental health [5]. Thus, improvement in surveillance systems, investments in technologies, and management practices in livestock are essential to impairing the spread of MDR bacteria [6].

Methicillin-resistant *Staphylococcus aureus* (MRSA) is an important MDR human pathogen in both healthcare and community settings [7]. It is considered a serious threat in terms of antibiotic resistance by the Centers for Disease Control and Prevention [8]. The World Health Organization also recognizes the importance of MRSA as a high-priority pathogen for which new therapies are urgently needed [9].

Over the past two decades, an increasing number of antimicrobial resistance studies have been conducted with *S. aureus* isolates associated with food-producing animals [10,11,12]. Certain MRSA genetic lineages, classified as livestock-associated MRSA (LA-MRSA), have emerged in these animals. CC398 (ST398) is the predominant LA-MRSA lineage in the world, but others have also been isolated, such as CC8, CC9, CC15, CC22, CC30, and CC97. CC398 is the most detected clone in livestock in Europe [13] and the USA [14], while CC9 is prevalent in Asia [15]. CC1 and CC5 lineages, commonly responsible for human infections, have also been recently recovered from animals. Studies have demonstrated transmission of these clones among humans, animals, and the environment [10,12]. The CC398 lineage has caused infections in humans, mainly in individuals with occupational exposure to livestock [16,17,18]. 

Evolutionary studies indicate that CC398 emerged among humans and subsequently underwent adaptations when infecting animals. The ancestral lineage was a methicillin-susceptible *S. aureus* (MSSA) strain that carried an immune evasion cluster (IEC), transferred by prophage Sa3int (φSa3), which is a host-specific marker of human strains. This cluster contains different combinations of highly human-specific virulence genes such as *sea*, *sep*, *sak*, *chp*, and *scn*. In animals, CC398 lost φSa3 and gained SCC*mec*, becoming MRSA, and tetracycline resistance genes. Later, MRSA CC398 spread in the livestock, mainly among pigs, but this lineage has also caused infections in humans [19].

MDR *S. aureus* colonization in pigs and farm workers may have an important role in the spread of resistant strains and resistance genes among animals, humans with direct animal contact, or even pork consumers, causing an impact on the treatment of possible infections. Brazil is a large producer, consumer, and exporter of pork [20], with different profiles of farms distributed across the country. The knowledge about the spread of MRSA strains able to cause human disease such as LA-MRSA is essential to driving approaches to prevent difficult-to-treat infections with increased lethality. In the present study, we investigated the presence of MDR *S. aureus* associated with colonization in pigs and farm workers and potential interspecies transmission in rural properties in the Rio de Janeiro state, Brazil. 

## 2. Results

### 2.1. Description of the Farms and Deographic Data of the Farm Workers

**Figure 1:**
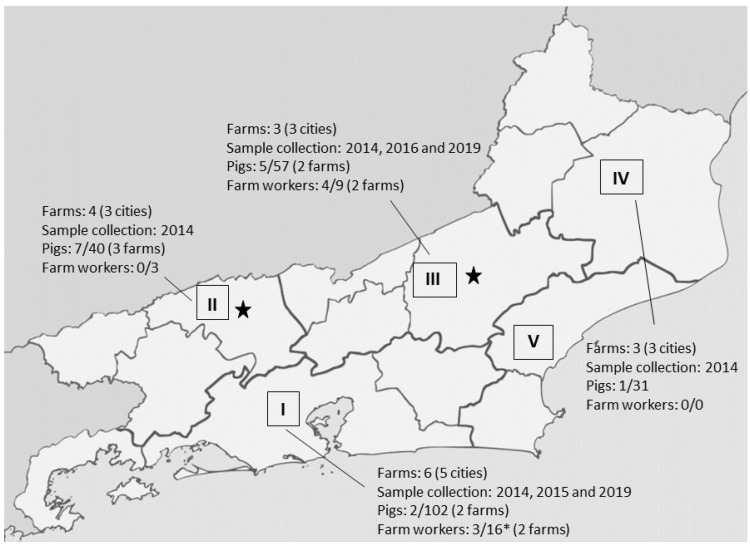
Distribution of the pig farms, pigs and farm workers investigated and colonized with *Staphylococcus aureus* by regions (I–V) of the Rio de Janeiro state from 2014 to 2019. Farms: C, G, K, M, O and P (I); A, B, D and E (II); F, L and N (III); H, I and J (IV). * Farms in regions I and II were attended by the same veterinarian, and his sample was collected only once. No sample collection was done in farms from cities of the region V. MRSA strains were recovered from pig farms located in cities from regions marked with a star (driving distance of 225 km between the cities). This figure was created from the map made by Allice Hunter—File: Brazil Rio de Janeiro location map.svg, CC BY-SA 4.0, https://commons.wikimedia.org/w/index.php?curid=70980877, accessed on 6 June 2024.

The pig farms investigated were small properties distributed in 14 cities located in four regions of the Rio de Janeiro state (Figure 1). Three farms (E, L, and N) sell their animals within the state, while the others sell them only in their own cities. Five farms (E, K, L, N, and O) used to clean their pens with chemical products and adopt downtime. Antimicrobial agents were used to treat pig infections in 12 (75%) farms (A–E, G–I, K, L, N, and O) and to treat pig infections and for prophylaxis in four (25%) of them (E, K, L, and N). Five (31.5%) farms (E, K, L, N, and O) used more than three antimicrobial agent classes. The drugs used include beta-lactams (n = 5), macrolides (n = 2), quinolones (n = 4), tetracycline (n = 9), aminoglycosides (n = 4), and sulfonamides (n = 3). 

The farm workers were animal handlers (n = 20), veterinarians or veterinary students (n = 6), and farmers (n = 3). Six workers had used antimicrobial agents (all beta-lactams) in the last six months. Only one worker had been hospitalized, and five workers lived with someone who had been hospitalized within a year before sample collection. Five workers lived with healthcare workers. Seventeen workers had occupational or non-occupational contact with other animals, such as cattle, horses, birds, goats, dogs, cats, and rabbits. Five employees work or had worked on other farms in the previous six months.

### 2.2. Pigs and Farm Workers Colonized with Multidrug-Resistant S. aureus 

*Staphylococcus aureus* was isolated from 14 (6.1%) of the 230 pigs distributed in eight farms of four regions (I–IV) in Rio de Janeiro state. Nine (60%) of these 15 pigs were colonized with MDR strains and belonged to five different farms (B, E, K, L, and N). In addition, *S. aureus* was isolated from seven (25.9%) of the 27 farm workers from four (K, L, N, and O) of the eleven farms analyzed. Five (71.4%) of these seven farm workers were colonized with MDR strains, which were detected in all four farms. In two farms, MRSA strains were recovered from pigs (farm E) or from pigs and farm workers (farm N) (Tables S1 and S2). Figure 1 shows the distribution of the farms, pigs, and farm workers by region.

### 2.3. Antimicrobial Resistance

Approximately 71.4% (10/14) and 86% (6/7) of the pigs and humans colonized with *S. aureus*, respectively, carried isolates that were resistant to at least one of the antimicrobial agents tested. Most animals were colonized with isolates resistant to clindamycin (n = 9), erythromycin (n = 9), ciprofloxacin (n = 9), penicillin (n = 9), and tetracycline (n = 9). Among humans, we found higher frequencies of colonization with isolates that were resistant to penicillin (n = 6), clindamycin (n = 5), tetracycline (n = 5), and erythromycin (n = 4). We did not detect linezolid and rifampicin resistance. Sulfamethoxazole-trimethoprim- and oxacillin-resistant isolates were obtained only from pigs, while isolates with inducible clindamycin resistance were only from humans (Table 1).

MRSA strains, identified by cefoxitin disk and PCR for *mecA*, were isolated from two pigs (SN51 and SN52) on farm E. However, oxacillin-susceptible *mecA*-positive (OS-MRSA) strains were recovered from another pig (SN18) and a farm worker (HSN182) on farm N. Therefore, the prevalence of MRSA colonization was 1.3% (3/230) in pigs and 3.7% (1/27) in farm workers. Regarding MDR strains, including MRSA and MSSA (methicillin-susceptible *S. aureus*), 3.9% (9/230) and 18.5% (5/27) of pigs and farm workers, respectively, were colonized with these bacteria. Among *S. aureus* carriers, 64.3% (9/14) of animals and 71.4% (5/7) of humans were colonized with MDR strains. Some animals and humans of the same farm carried *S. aureus* strains that exhibited similar or indistinguishable resistance patterns (Table S2). 

MDR strains were recovered from pigs in five (31.3%; B, E, K, L, and N) of the 16 farms, and in four (80%; E, K, L, and N) of them, chemical disinfection of the pens and downtime were carried out, antimicrobial agents were used for prophylaxis and treatment, and at least three classes of antimicrobial agents were used. These management practices, except for the use of antimicrobial agents for prophylaxis, were also adopted by one (9.1%; O) of the farms where no colonization with MDR *S. aureus* was detected among the animals investigated. MDR *S. aureus* strains were isolated from animals in the three largest farms and with the widest commercialization area (E, L, and N). 

Among the farm workers colonized with MDR *S. aureus*, one was a veterinary student (20%; HSN10), one was a veterinarian (20%; HSN21), and three were animal handlers (60%; HSN12, HSN16, and HSN18). Two farm workers (40%; HSN12 and HSN21) used antimicrobial agents in the last six months; one (20%; HSN21) of them had contact with a hospitalized individual and worked on another rural property. Three (13.6%) of the twenty-two non-colonized workers had used antimicrobial agents in the last six months, one (4.6%) farm worker had been hospitalized in the previous year, four (18.2%) farm workers had had contact with a hospitalized individual, five (22.7%) farm workers lived with a healthcare professional, and 14 (63.6%) farm workers had contact with other animals.

### 2.4. Genomic Characterization of MDR Strains

Five MDR strains were selected for whole genome sequencing (WGS) analysis: SN51 (MRSA, farm E), SN145 and HSN12 (MSSA, farm L), and SN182 and HSN18 (OS-MRSA, farm N). Three of them were LA-MRSA ST398-SCCmecV-t011: two were isolated from pigs (SN51 and SN182) and one was isolated from a farm worker (HSN18), with a pair of pig–farm worker from the same farm. The other two strains were LA-MSSA ST-398, isolated from one pig (SN145) and one farm worker (HSN12) in farm L, and exhibited closely related *spa* types (t571: 08-16-02-25-02-25-34-25; t1451: 08-16-02-25-34-25). 

The pig and human strains recovered from the same farm also shared the same resistome. Resistance genes were detected for beta-lactams (*blaZ*, *mecA*), tetracycline [*tet*(M), *tet*(K), and *tet*(L)], amphenicols (*fex*), macrolides [*erm*(C), *erm*(T)], lincosamides [*erm*(C), *erm*(T), *lsa*(E)], streptogramin B [*lsa*(E)], and trimethoprim (*dfr*). Mutations (*gyrA*: S84L and *grlA*: S80Y) that confer resistance to quinolones were also detected. Aminoglycoside resistance genes [*aac*(6”)-*aph*(2”) and *aaD*] were found only in the MSSA isolates. WGS analysis confirmed the presence of the mecA gene in strains that were susceptible to oxacillin (cefoxitin disk), but *mecA*-positive by PCR. The *qacG* gene, which confers resistance to quaternary ammonium compounds (QAC), was also detected in the strains, except for SN51.

Four virulence genes (*aur*, *hlgA*, *hlgB*, and *hlgC*) were detected, and all strains exhibited the same profile, except one from a farm worker (HSN12). The aur gene encodes aureolysin (Aur), a metalloprotease, and the *hlgA, hlgB*, and *hlgC* genes encode gamma-hemolysin AB (Hlg AB) and gamma-hemolysin CB (Hlg CB), bicomponent *pore*-*forming* leucocidins. In the five strains, several mobile genetic elements (MGEs) were detected, and some of them were common to all, such as plasmid repUS43 and integrative conjugative elements (ICE) Tn558 and Tn6009. Identical or similar backgrounds were shared among strains from pigs and farm workers in the farms. Prophage φSa3 was not found in any of the strains. Data obtained by WGS analyses are shown in Table 2.

### 2.5. Phylogenetic Analyses

#### 2.5.1. Core Genome Single-Nucleotide Polymorphism (cgSNP) Analysis

**Figure 2:**
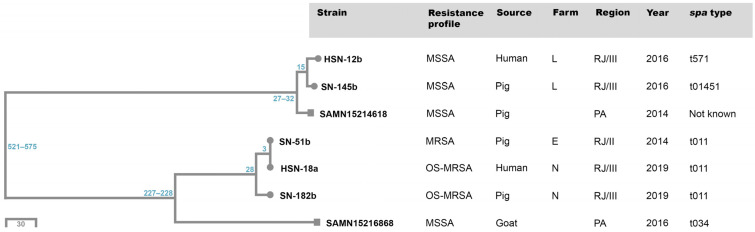
Single nucleotide polymorphism (SNP)-based neighbor-joining tree generated using the Pathogenwatch web application based on core genome of seven *Staphylococcus aureus* ST398 strains from human and animal source in Brazil. The five study multidrug-resistant strains (circle) are presented together with two Brazilian *S. aureus* isolates (square) available at Pathogenwatch website (SAMN15214618 from swine nasal swab and SAMN15216868 from goat milk). SNP differences between strains are shown in blue.

Using the Pathogenwatch web application, core matches among the five study MDR strains selected for WGS varied from 1610 to 1627 genes, with 1578 to 1614 complete alleles, making up 99% to 99.8% of the core families. Genomes of the two additional strains from Northeastern Brazil available at the Pathogenwatch database had 1625 and 1627 core matches, with 1609 and 1614 complete alleles, making up 99.8% and 99.9% of the core families. SNP divergences based on the core genome between all the seven isolates included in the cgSNP analysis varied from 3 to 575. Two clusters were clearly observed, showing a close relationship between strains from different hosts, farms, and years (Figure 2).

#### 2.5.2. Core Genome Multilocus Sequence Typing (cgMLST)

Of 1861 genes of the Ridom ™ SeqSphere+ cgMLST scheme for *S. aureus*, 170 genes with missing values in at least one strain were removed from the analysis. Allele assignment varied from 1758 (94.5%) to 1816 (97.6%) per isolate. Two clusters were formed. One cluster had three study strains: HSN18a, SN51b, and SN182b. Strains HSN18a and SN51b had identical cgMLST profiles, and they differed from strain SN182b in 16 alleles. The second cluster comprised two study strains (HSN12b and SN145b), which differed from each other in 13 alleles. The distance between the two clusters varied from 230 to 235 alleles. The strain SAMN15214618, from the Pathogenwatch database, had 26–27 allele differences from strains HSN12b and SN145b, but it was not included in the same cluster since the maximum distance of 24 alleles defines a complex type. The complex type of the Pathogenwatch strain SAMN15216868 was defined as 35475, and this strain differed from the others in 174 to 273 alleles.

## 3. Discussion

Our study presents data on MDR *S. aureus* colonization of pigs and people working on farms in the Rio de Janeiro state. We detected both animals and humans from the same farms colonized with MDR *S. aureus* strains, including LA-MRSA ST398 t011. This lineage was shared by one pig and one human from the same farm and was also isolated from a pig on another farm in a different city (with a distance of 225 km), suggesting interspecies transmission and a potential distribution of this lineage in different farms in the Rio de Janeiro state. The presence of LA-MRSA ST398 t011 in these two farms can be implicated in different epidemiological settings, such as sporadic occurrence, onset of an outbreak, or endemic presence of this strain. Nevertheless, our study was not designed to answer this question. 

Colonization prevalence with *S. aureus* in pigs is highly variable (0% to 77%), but generally elevated frequencies have been observed [21,22,23,24,25]. Discrepant prevalence may be due to the collection of samples from more than one body site or the surveyed farm profile. Here, the low colonization prevalence of pigs (6%) may be related to the predominance of small farms investigated. A higher colonization proportion was observed (24%) among humans compared to pigs, despite the analysis of fewer farm workers. This colonization frequency is similar to those observed in studies with the general human population [22,26].

Although a few pigs have been colonized with *S. aureus*, MDR (either MRSA or MSSA) strains were found in most of them, mainly in farms that used a greater variety of antimicrobial agents and for purposes other than treatment. In these farms, similar resistance phenotypic profiles were observed among strains obtained from pigs and humans, suggesting interspecies transmission. Many isolates showed resistance to up to nine antimicrobial classes, regardless of the host species. As in other countries, high resistance frequencies were observed to antimicrobial agents commonly used in pig farms, with frequency variations according to the geographic region [27,28]. 

In our study, MRSA colonization was observed in 1.2% and 3.5% of pigs and farm workers. The frequency of MRSA colonization has been variable among pigs in several countries. In some studies, MRSA was not detected despite the high frequency of *S. aureus* isolation from animals [21,22]. In others, the frequencies reported exceeded 50% [28,29]. In relation to pig farm workers, variable frequencies have been observed, either similar or higher than those observed for the general population [28,30]. Two oxacillin-susceptible strains were identified as OS-MRSA by detection of *mecA*. OS-MRSA strains have been reported both in humans [31,32] and animals [33,34] in several countries, with variable prevalence. In Brazil, these strains have already been isolated from dogs [34], cattle [33], and humans [35,36].

WGS analyses of five MDR strains showed pigs and farm workers colonized with strains of identical ST, identical or related *spa* types, and identical resistance genetic background. All MRSA strains, recovered from two pigs and one human, belonged to ST398, t011, and carried the *mec*A gene and the SCC*mec* type V. The other strains, also recovered from both host species, were MSSA ST398 and had different *spa* types, although related to each other (t571 and t01451). The *spa* type t011 is the most found among LA-MRSA ST398 strains in European countries and the United States [22,37,38]. Other *spa* types, such as t571 and t01451, have also been reported among ST398 strains in these regions [28,39] and Korea [25,40]. In Brazil, the first report of detection of ST398 in pigs was from exudative epidermitis [41]. This strain was a vancomycin-intermediate LA-MRSA ST398/t9538. More recently, ST398/t571 and t1471 strains resistant to oxacillin, but lacking *mecA* or its variants, were isolated from healthy pigs from two farms in Northeastern Brazil [42]. 

All MDR strains contained the *tet*(M) gene and lacked the φSa3 phage, which are characteristics of the livestock-associated CC398 clade. The *dfrG* gene was also detected in all strains sequenced in our study. This gene, common pig-associated clade, encodes a dihydrofolate reductase (DHFR) variant, which confers lower affinity to trimethoprim [16,19,43].

Multidrug resistance genes, such as *lsa*(E), which confers resistance to lincosamides, pleuromutilin, and streptogramin A, and *erm*, which confers resistance to macrolides, lincosamides, and streptogramin B (MLS_B_), were also found. The *lsa*(E) gene encodes an ABC transporter and has been detected in LA-MRSA ST9 and ST398 [44,45,46]. The *erm* genes encode an rRNA methyltransferase that modifies the target of MLSB antimicrobial agents. Among these genes, *erm*(C) is the most widespread among staphylococci [47,48], including LA-MRSA ST398 [49]. In our study, *erm*(C) was detected in the LA-MRSA ST398 strains, while *erm*(T) was found in the LA-MSSA ST398 strains. The *erm*(T) gene has been found most frequently in human-associated ST398 strains [16]. 

Other tetracycline resistance genes were also detected in all strains, such as *tet*(K) or *tet*(L), which encode efflux pumps. Differently, the *tet*(M) gene confers another action mechanism that consists in ribosomal protection. Here, LA-MSSA ST398 strains were resistant to aminoglycoside, and LA-MRSA ST398 were resistant to quinolone. The detected aminoglycoside resistance genes were *aac*(6’)-*aph*(2”) and *aadD*, which encode modifying enzymes. Quinolone-resistant strains had mutations in *gyrA*(S84L) or *glrA*(S80Y). The *tet*(K), *aac*(6*’*)*-aph*(2”), and *aadD* genes have also been commonly reported among LA-MRSA CC398 strains, as well as mutations in *gyrA*(S84L) [49,50].

Finally, all chloramphenicol-resistant strains carried the *fexA* gene, which encodes an efflux protein. The most common resistance mechanism is the production of chloramphenicol acetyltransferase (CAT) enzymes. Differently from the *fexA*-encoding efflux protein, CAT enzymes have no activity against florfenicol, which is one antimicrobial drug used in veterinary medicine [51]. Probably, florfenicol use may be related to selection of this gene among *S. aureus* in livestock. 

All five MDR were negative for *luk*SF-PV genes, which encode the Panton-Valentine leucocidin (PVL). This toxin has been associated with some genetic lineages of community-acquired MRSA. Other studies have not detected the *luk*SF-PV genes among LA-MRSA ST398 either [10,52]. 

Based on different core genome analyses, we have also detected potential interspecies transmission of MDR strains. SNP rates among *S. aureus* strains usually vary from 3.4 to 13 SNPs/genome/year. A recent study with MRSA carriers found a median within-host mutation rate in long-term colonization of 4.9 (3.4–6.9) SNPs/genome/year [53]. Goyal et al. (2019) [54] demonstrated that the pairwise SNP distances between *S. aureus* strains were 0 to 5 SNPs (median 2) during a 1-month period of artificial colonization in human volunteers. In addition, core genome pairwise SNP divergence between *S. aureus* strains ranged from 9 to 57 SNPs (median 20) over a period of 3 years of natural colonization in persistent human carriers. Taking into account these mutation rates, we can suggest that strains HSN18a, SN51b, and SN182b may have been transmitted between different hosts and farms in a short period of time. Similar estimates may be done for strains HSN12b and SN145b. The cgMLST analysis confirmed the close relationship of the study MDR strains, with the observation of two well-defined clusters. One strain from a swine nasal swab from Northeastern Brazil (with a distance of over 2000 km from the study sites) was shown to be closely related to two study strains isolated from pigs and humans in the cgSNP analysis; however, the cgMLST approach did not confirm this finding. 

Analysis of the potential association between management practices and MDR *S. aureus* colonization was not possible due to a small sample size. However, most of the farms where MDR *S. aureus* were isolated from pigs carried out disinfection of pens and downtime, used at least three classes of antimicrobial agents, and used antimicrobial agents for purposes other than treatment. A broader use of antimicrobial agents is expected to be related to greater MDR colonization in pigs. Use of disinfectants and downtime are biosecurity practices adopted to reduce the introduction and dissemination of infectious agents among animals. Nevertheless, disinfectant resistance may contribute to the selection of MDR bacteria, especially when the responsible genes are carried along with antimicrobial resistance genes in MGE [55]. In our study, MDR strains carrying one gene that confers resistance to QAC, compounds widely used in swine farming, were isolated from two farms. The application of downtime between animal batches may not have an impact on the load of certain microorganisms in the pens, depending on disinfection effectivity. Luyckx et al. (2016) showed that even with a longer downtime period, some bacteria can survive in the environment, such as MRSA, *Enterococcus*, and *Escherichia coli* [56]. Another hypothesis for the perpetuation of animals colonized with MDR in farms with efficient disinfection and downtime would be through interspecies transmission. Colonized farm workers could transmit these bacteria to animals. Thus, studies with a larger sample size should be carried out to identify potential associations. 

Limitations of the study include the low number of animals and especially of farm workers evaluated, the absence of human samples from all properties, and the heterogeneous profile of farms analyzed, which included mainly small and local producers. Nevertheless, the finding of LA-MRSA ST398 occurrence among the investigated animal and human participants is of great relevance and concern.

## 4. Materials and Methods

### 4.1. Study Design and Period 

The study was performed in two cross-sectional periods, from January 2014 to November 2016 in 12 farms and from May 2019 to November 2019 in four farms in the Rio de Janeiro state (Southeastern Brazil) (Table S1).

### 4.2. Setting 

The farms are located in 14 different cities with driving distances from 24 to 478 km. Rio de Janeiro state has 92 cities grouped into five geographic regions here designated by a roman number (I–V), each influenced by a representative urban center [57]. The pig farms were selected in the way that different regions were investigated (Figure 1). Most farms were small properties, and their production is commercialized in their own city or in other cities of the Rio de Janeiro state. 

### 4.3. Sample Collection

Nasal swabs were collected from 230 pigs to research the presence of colonization with MDR *S. aureus* and to identify the MDR genetic lineages circulating in this region. We prioritized the collection of swabs from pigs that were in different pens of each farm. Farm worker samples were collected to detect the presence of MDR *S. aureus* and to compare with pig strains to identify close related lineages and potential interspecies cross-transmission in the farms. The consent from employees was not obtained in all farms; thus, samples of these individuals were not collected. Then, nasal swabs were collected from 27 workers from eleven farms (A–E, K–P) (Table S1). Farm workers were veterinarians or veterinary students (n: 6), employees who were taking care of the animals (n: 20), and farm managers or owners (n: 3). For all farms, nasal swabs were collected in a single visit. All swabs were placed into Stuart medium at 4 °C and transported for further processing in the laboratory within five days of sample collection. We also used a questionnaire to collect data on farm management practices and individuals. to identify possible factors associated with colonization with MDR *S. aureus*. In the farm questionnaire, data were obtained on commercialization area, cleaning of pens, adoption of downtime, and use of antimicrobial agents [reason for use (treatment and/or prophylaxis) and drugs used]. 

### 4.4. Demogratphics and Management Pratices Data

The data collected from farm workers were age, sex, race, occupation on the farm, presence of skin lesions, recent use of antimicrobials and which antimicrobial agent, previous hospitalization, contact with a hospitalized person, residence with a healthcare worker and contact with other animals. 

### 4.5. Bacterial Isolation and Identification 

After isolation on mannitol salt agar (BD, Sparks, MD, USA), with and without oxacillin (2 µg/mL) (Sigma, St. Louis, MO, USA), up to five colonies were selected per animal and human and initially identified by Gram staining, catalase, and tube coagulase tests. Gram-positive, catalase-positive, and coagulase-positive cocci were further subjected to MALDI-TOF mass spectrometry for species identification (matrix-assisted laser desorption ionization–time of flight) in a Microflex LT instrument (Bruker Daltonik, Bremen, Fahrenheitstraße, Germany) [58].

### 4.6. Antimicrobial Susceptibility Testing 

Antimicrobial resistance was determined by the disk-diffusion method. Antimicrobial agents tested were cefoxitin (30 µg), chloramphenicol (30 µg), ciprofloxacin (5 µg), clindamycin (2 µg), erythromycin (15 µg), gentamicin (10 µg), linezolid (30 µg), penicillin (10 U), rifampin (5 µg), sulfamethoxazole/trimethoprim (1.25/23.75 μg), and tetracycline (30 µg) (Cecon, São Paulo, SP, 96 Brazil). Inducible clindamycin resistance was investigated by disk approximation test (D test). MRSA strains were tested for vancomycin susceptibility by broth microdilution [59]. In addition to the cefoxitin disk, we tested all isolates by PCR targeting the *mecA* gene to detect MRSA strains. In *mecA*-positive strains, the type of SCC*mec* was also determined [60]. MRSA strains and strains non-susceptible to at least one agent in three or more antimicrobial classes were considered MDR [61].

### 4.7. Whole Genome Sequencing (WGS) for Characterization of MDR Isolates 

Five MDR strains were subjected to WGS analysis. We selected MRSA strains as well as strains with the same antimicrobial resistance profile observed in pigs and humans from the same farm to identify which MRSA genetic lineages were circulating and investigate potential transmission between animals and farm workers, respectively. Only one MRSA strain was chosen when MRSA strains with similar resistance profile and molecular characteristics (assessed by PCR for *mecA, pvl*, and SCC*mec* types) were isolated from more than one animal that shared the same pen at the time of collection. WGS was performed by the BPI Biotechnology sequencing facility (Bauru, Brazil) or MicrobesNG (Birmingham, UK) using the Illumina NovaSeq platform. The sequence type (ST) was determined on the PubMLST website. We used spaTyper 1.0 and SCCmecFinder 1.2 tools to identify the *spa* type and the SCC*mec* type, respectively. Analysis of antimicrobial resistance genetic content was performed with ResFinder 4.1 and MobileElementFinder. The presence of virulence genes was investigated with VirulenceFinder 2.0. All these tools are available on the Center for Genomic Epidemiology website (https://www.genomicepidemiology.org/services/, accessed on 15 September 2021). Prophage φSa3 was researched in the genome sequences with PHASTEST (https://phastest.ca/, accessed on 24 May 2024).

All five MDR strains were also subjected to core genome analyses. A core-genome tree was constructed based on single nucleotide polymorphism (cgSNP analysis) distance and neighbor-joining method. Core assignment, reference assignment, core filtering, and tree construction were done using the Pathogenwatch web application (https://cgps.gitbook.io/pathogenwatch/technical-descriptions/core-genome-tree, accessed on 26 May 2024). In addition, we used the Ridom™ SeqSphere+ (Version 10.0.0) cgMLST scheme for *S. aureus* with 1861 core genes for strain clustering. Core genes with failure in allele assignment due to not detection or incompleteness were removed before calculating a distance matrix. Strains with a maximum distance of 24 alleles were considered to belong to the same complex type (https://www.cgmlst.org/ncs/schema/141106/, accessed on 26 May 2024). Two *S. aureus* ST398 strains available at the Pathogenwatch website database, recovered from pig nasal swabs in 2014 (SAMN15214618) and goat milk in 2016 (SAMN15216868) from Paraíba state (Northeastern Brazil), were included in the analysis for comparative purposes.

### 4.8. Statistical Analysis

Analyses of categorical variables were performed with absolute numbers and proprotions with EpiInfo version 7.2 (https://www.cdc.gov/epiinfo/support/por/pt_downloads.html, accessed on 26 May 2024). 

## 5. Conclusions

In conclusion, we detected colonization with MDR *S. aureus* strains in pigs and farm workers in different farms of the Rio de Janeiro state, Brazil. LA-MRSA ST398 and LA-MSSA ST398 carrying a wide variety of antimicrobial resistance genes were shown to circulate among pigs and rural workers in these farms. Moreover, different hosts within the same farm shared strains with identical genetic backgrounds, suggesting potential interspecies transmission. Further studies are necessary to investigate factors associated with MDR *S. aureus* colonization and interspecies transmission in the region investigated. 

## Figures and Tables

**Table 1:** Frequency of carriers of strains resistant to different antimicrobial agents among pigs and farm workers colonized with *Staphylococcus aureus*.

Antimicrobial Agent	Number of Carriers of Resistant Strains	
	Pig (14)	Farm Worker (7)
Clindamycin	9	5
Erythromycin	9	4
Chloramphenicol	7	2
Penicillin	9	6
Tetracycline	9	5
Ciprofloxacin	9	3
Norfloxacin	6	1
Sulfamethoxazole-trimethoprim	5	0
Gentamycin	3	2
Cefoxitin	2	0
Linezolid	0	0
Rifampicin	0	0

Cefoxitin disk identified MRSA (methicillin-resistant *Staphylococcus aureus*) strains.

**Table 2:** Characteristics of five MDR *Staphylococcus aureus* strains recovered from pigs and farm workers obtained by disk diffusion and whole-genome sequencing analyses.

Host	Source	Farm	ST	*spa* Type	SCCmec	Resistance		Virulence Genotype	Plasmids	IS, ICE
						Phenotype ^1^	Genotype			
HSN12	Human	L	398	t571	-	Chl, Cip, Cli, Ery, Gen, Nor, Pen, Tet	*blaZ*, *aac*(6”)-*aph*(2”), *aaD*, *erm*(T), *lsa*(E), *tet*(L), *tet*(M), *fexA*, *dfrG*, *qacQ*	*aur*, *hlgA*	rep21, rep22, repUS43, repUS70	IS*256*, IS*Sau1*, IS*Sau8*, Tn*558*, Tn*6009*
SN145	Pig	L	398	t01451	-	Chl, Cip, Cli, Ery, Gen, Nor, Pen, Tet	*blaZ*, *aac*(6”)-*aph*(2”), *aaD*, *erm*(T), *lsa*(E), *tet*(L), *tet*(M), *fexA*, *dfrG*, *qacQ*	*aur*, *hlgA*, *hlgB*, *hlgC*	rep21, rep22, repUS43, repUS70	IS*256*, IS*Sau1*, Tn*558*, Tn*6009*
SN51^2^	Pig	E	398	t011	V	Cef, Cip, Cli, Ery, Nor, Pen, Sut, Tet	*bla*Z, *mecA*, *erm*(C), *lsa*(E), *tet*(K), *tet*(M), *gyrA*, *fexA*, *dfrG*	*aur*, *hlgA*, *hlgB*, *hlgC*	rep7a, repUS43	Tn*558*, Tn*6009*
HSN18^3^	Human	N	398	t011	V	Chl, Cip, Cli, Ery, Pen, Tet	*bla*Z, *mecA*, *erm*(C), *lsa*(E), *tet*(K), *tet*(M), *grlA*, *dfrG*, *qacQ*	*aur*, *hlgA*, *hlgB*, *hlgC*	rep7a, repUS43	Tn*558*, Tn*6009*
SN182^3^	Pig	N	398	t011	V	Chl, Cip, Cli, Ery, Pen, Tet	*bla*Z, *mecA*, *erm*(C), *lsa*(E), *tet*(K), *tet*(M), *grlA*, *dfrG*, *qacQ*	*aur*, *hlgA*, *hlgB*, *hlgC*	rep7a, repUS43	Tn*558*, Tn*6009*

^1^ Cef: cefoxitin; Cip: ciprofloxacin, Chl: chloramphenicol; Cli: clindamycin, Ery: erythromycin, Gen: gentamicin, Nor: norfloxacin, Pen: penicillin G, Sut: sulfamethoxazole-trimethoprim, Tet: tetracycline, ^2^ MRSA (methicillin-resistant *S. aureus*), ^3^ OS-MRSA (oxacillin-susceptible MRSA).

## Data Availability

The genomes of the strains included in this study are available in the GenBank (BioProject ID: PRJNA1143536) databases.

## Supplementary Materials

The following supporting information can be downloaded at: https://www.mdpi.com/article/10.3390/antibiotics13080767/s1, Table S1: Number of samples collected and of pigs and farm workers colonized with *S. aureus* in each farm investigated in the study. Table S2: Antimicrobial resistance patterns of multidrug-resistant *Staphylococcus aureus* strains from pigs and farm workers in pig farms from Rio de Janeiro state.

## References

[1] Shi X., Wang S. (2018). Antibiotic resistance in environment of animal farms. Chin. J. Biotechnol..

[2] Muurinen J., Richert J., Wickware C.L., Richert B., Johnson T.A. (2021). Swine growth promotion with antibiotics or alternatives can increase antibiotic resistance gene mobility potential. Sci. Rep..

[3] Barrett J.R., Innes G.K., Johnson K.A., Lhermie G., Ivanek R., Greiner Safi A., Lansing D. (2021). Consumer perceptions of antimicrobial use in animal husbandry: A scoping review. PLoS ONE.

[4] Innes G.K., Markos A., Dalton K.R., Gould C.A., Nachman K.E., Fanzo J., Barnhill A., Frattaroli S., Davis M.F. (2021). How animal agriculture stakeholders define, perceive, and are impacted by antimicrobial resistance: Challenging the Wellcome Trust’s Reframing Resistance principles. Agric. Hum. Values.

[5] McEwen S.A., Collignon P.J. (2018). Antimicrobial resistance: A One Health perspective. Microbiol. Spectr..

[6] Musoke D., Namata C., Lubega G.B., Niyongabo F., Gonza J., Chidziwisano K., Nalinya S., Nuwematsiko R., Morse T. (2021). The role of environmental health in preventing antimicrobial resistance in low- and middle-income countries. Environ. Health Prev. Med..

[7] Otto M. (2013). Community-associated MRSA: What makes them special?. Int. J. Med. Microbiol..

[8] CDC (2019). Antibiotic Resistance Threats in the United States.

[9] WHO (2024). Bacterial Priority Pathogens List, 2024: Bacterial Pathogens of Public Health Importance to Guide Research, Development and Strategies to Prevent and Control Antimicrobial Resistance.

[10] Back S.H., Eom H.S., Lee H.H., Lee G.Y., Park K.T., Yang S.J. (2020). Livestock-associated methicillin-resistant *Staphylococcus aureus* in Korea: Antimicrobial resistance and molecular characteristics of LA-MRSA strains isolated from pigs, pig farmers, and farm environment. J. Vet. Sci..

[11] Cheng W.N., Han S.G. (2020). Bovine mastitis: Risk factors, therapeutic strategies, and alternative treatments—A review. Asian-Australas J. Anim. Sci..

[12] Wang Y., Zhang P., Wu J., Chen S., Jin Y., Long J., Duan G., Yang H. (2023). Transmission of livestock-associated methicillin-resistant *Staphylococcus aureus* between animals, environment, and humans in the farm. Environ. Sci. Pollut. Res. Int..

[13] Avberšek J., Golob M., Papić B., Dermota U., Grmek Košnik I., Kušar D., Ocepek M., Zdovc I. (2021). Livestock-associated methicillin-resistant *Staphylococcus aureus*: Establishing links between animals and humans on livestock holdings. Transbound. Emerg. Dis..

[14] Smith T.C., Thapaliya D., Bhatta S., Mackey S., Engohang-Ndong J., Carrel M. (2018). Geographic distribution of livestock-associated *Staphylococcus aureus* in the United States. Microbes Infect..

[15] Fang H.W., Chiang P.H., Huang Y.C. (2014). Livestock-associated methicillin-resistant *Staphylococcus aureus* ST9 in pigs and related personnel in Taiwan. PLoS ONE.

[16] Bouiller K., Bertrand X., Hocquet D., Chirouze C. (2020). Human infection of methicillin-susceptible *Staphylococcus aureus* CC398: A review. Microorganisms.

[17] Chen H., Yin Y., Li X., Li S., Gao H., Wang X., Zhang Y., Liu Y., Wang H. (2020). Whole-genome analysis of livestock-associated methicillin-resistant *Staphylococcus aureus* sequence type 398 strains isolated from patients with bacteremia in China. J. Infect. Dis..

[18] Abdullahi I.N., Lozano C., Zarazaga M., Simón C., Höfle U., Sieber R.N., Latorre-Fernández J., Stegger M., Torres C. (2024). Comparative genomics of *Staphylococcus aureus* strains from wild birds and pig farms elucidates levels of mobilomes, antibiotic pressure and host adaptation. J. Glob. Antimicrob. Resist..

[19] Price L.B., Stegger M., Hasman H., Aziz M., Larsen J., Andersen P.S., Pearson T., Waters A.E., Foster J.T., Schupp J. (2012). *Staphylococcus aureus* CC398: Host adaptation and emergence of methicillin resistance in livestock. mBio.

[20] Rabello R.F., Bonelli R.R., Penna B.A., Albuquerque J.P., Souza R.M., Cerqueira A.M.F. (2020). Antimicrobial resistance in farm animals in brazil: An update overview. Animals.

[21] Burns A., Shore A.C., Brennan G.I., Coleman D.C., Egan J., Fanning S., Galligan M., Gibbons J., Gutierrez M., Malhotra-Kumar S. (2014). A longitudinal study of *Staphylococcus aureus* colonization in pigs in Ireland. Vet. Microbiol..

[22] Sun J., Yang M., Sreevatsan S., Davies P.R. (2015). Prevalence and characterization of *Staphylococcus aureus* in growing pigs in the USA. PLoS ONE.

[23] Nobrega D.B., De Buck J., Naqvi S.A., Liu G., Naushad S., Saini V., Barkema H.W. (2017). Comparison of treatment records and inventory of empty drug containers to quantify antimicrobial usage in dairy herds. J. Dairy Sci..

[24] Velasco V., Vergara J.L., Bonilla A.M., Muñoz J., Mallea A., Vallejos D., Quezada-Aguiluz M., Campos J., Rojas-García P. (2018). Prevalence and characterization of *Staphylococcus aureus* strains in the pork chain supply in Chile. Foodborne Pathog. Dis..

[25] Eom H.S., Back S.H., Lee H.H., Lee G.Y., Yang S.J. (2019). Prevalence and characteristics of livestock-associated methicillin-susceptible *Staphylococcus aureus* in the pork production chain in Korea. J. Vet. Sci..

[26] Sakr A., Brégeon F., Mège J.L., Rolain J.M., Blin O. (2018). *Staphylococcus aureus* nasal colonization: An update on mechanisms, epidemiology, risk factors, and subsequent infections. Front. Microbiol..

[27] Reynaga E., Navarro M., Vilamala A., Roure P., Quintana M., Garcia-Nuñez M., Figueras R., Torres C., Lucchetti G., Sabrià M. (2016). Prevalence of colonization by methicillin-resistant *Staphylococcus aureus* ST398 in pigs and pig farm workers in an area of Catalonia, Spain. BMC Infect. Dis..

[28] Abreu R., Rodríguez-Álvarez C., Lecuona M., Castro B., González J., Aguirre-Jaime A., Arias Á. (2019). Increased antimicrobial resistance of MRSA strains isolated from pigs in Spain between 2009 and 2018. Vet. Sci..

[29] Alt K., Fetsch A., Schroeter A., Guerra B., Hammerl J.A., Hertwig S., Senkov N., Geinets A., Mueller-Graf C., Braeunig J. (2011). Factors associated with the occurrence of MRSA CC398 in herds of fattening pigs in Germany. BMC Vet. Res..

[30] Armand-Lefevre L., Ruimy R., Andremont A. (2005). Clonal comparison of *Staphylococcus aureus* isolates from healthy pig farmers, human controls, and pigs. Emerg. Infect. Dis..

[31] Pu W., Su Y., Li J., Li C., Yang Z., Deng H., Ni C. (2014). High incidence of oxacillin-susceptible *mecA*-positive *Staphylococcus aureus* (OS-MRSA) associated with bovine mastitis in China. PLoS ONE.

[32] Saeed K., Ahmad N., Dryden M., Cortes N., Marsh P., Sitjar A., Wyllie S., Bourne S., Hemming J., Jeppesen C. (2014). Oxacillin-susceptible methicillin-resistant *Staphylococcus aureus* (OS-MRSA), a hidden resistant mechanism among clinically significant isolates in the Wessex region/UK. Infection.

[33] Guimarães F.F., Manzi M.P., Joaquim S.F., Richini-Pereira V.B., Langoni H. (2017). Short communication: Outbreak of methicillin-resistant *Staphylococcus aureus* (MRSA)-associated mastitis in a closed dairy herd. J. Dairy Sci..

[34] Fabri F.V., Pinto N.B., Mattos M.D.S.F.D., Rodrigues R.F., Shinohara D.R., Pereira P.M., Nishiyama S.A.B., Tognim M.C.B. (2021). First report of oxacillin-susceptible *mecA*-positive *Staphylococcus aureus* in healthy dogs and their owners in southern Brazil. Prev. Vet. Med..

[35] Andrade-Figueiredo M., Leal-Balbino T.C. (2016). Clonal diversity and epidemiological characteristics of *Staphylococcus aureus*: High prevalence of oxacillin-susceptible *mecA*-positive *Staphylococcus aureus* (OS-MRSA) associated with clinical isolates in Brazil. BMC Microbiol..

[36] Danelli T., Duarte F.C., Oliveira T.A.D., Silva R.S.D., Frizon Alfieri D., Gonçalves G.B., de Oliveira C.F., Tavares E.R., Yamauchi L.M., Perugini M.R.E. (2020). Nasal carriage by *Staphylococcus aureus* among healthcare workers and students attending a university hospital in Southern Brazil: Prevalence, phenotypic, and molecular characteristics. Interdiscip. Perspect. Infect. Dis..

[37] Denis O., Suetens C., Hallin M., Catry B., Ramboer I., Dispas M., Willems G., Gordts B., Butaye P., Struelens M.J. (2009). Methicillin-resistant *Staphylococcus aureus* ST398 in swine farm personnel, Belgium. Emerg. Infect. Dis..

[38] De Neeling A.J., Van Den Broek M.J.M., Spalburg E.C., Van Santen-Verheuvel M.G., Dam-Deisz W.D.C., Boshuizen H.C., van de Giessen A.W., van Duijkeren E., Huijsdens X.W. (2007). High prevalence of methicillin resistant Staphylococcus aureus in pigs. Vet. Microbiol..

[39] Wardyn S.E., Stegger M., Price L.B., Smith T.C. (2018). Whole-genome analysis of recurrent *Staphylococcus aureus* t571/ST398 infection in farmer, Iowa, USA. Emerg. Infect. Dis..

[40] Moon D.C., Jeong S.K., Hyun B.H., Lim S.K. (2019). Prevalence and characteristics of methicillin-resistant *Staphylococcus aureus* isolates in pigs and pig farmers in Korea. Foodborne Pathog. Dis..

[41] Moreno L.Z., Dutra M.C., Moreno M., Ferreira T.S., Silva G.F.D., Matajira C.E., Silva A.P., Moreno A.M. (2016). Vancomycin-intermediate livestock-associated methicillin-resistant *Staphylococcus aureus* ST398/t9538 from swine in Brazil. Mem. Inst. Oswaldo Cruz.

[42] Santos S.C.L., Saraiva M.M.S., Moreira Filho A.L.B., Silva N.M.V., De Leon C.M.G., Pascoal L.A.F., Givisiez P.E.N., Gebreyes W.A., Oliveira C.J.B. (2021). Swine as reservoirs of zoonotic borderline oxacillin-resistant *Staphylococcus aureus* ST398. Comp. Immunol. Microbiol. Infect. Dis..

[43] Nurjadi D., Olalekan A.O., Layer F., Shittu A.O., Alabi A., Ghebremedhin B., Schaumburg F., Hofmann-Eifler J., Van Genderen P.J., Caumes E. (2014). Emergence of trimethoprim resistance gene *dfrG* in *Staphylococcus aureus* causing human infection and colonization in sub-Saharan Africa and its import to Europe. J. Antimicrob. Chemother..

[44] Li B., Wendlandt S., Yao J., Liu Y., Zhang Q., Shi Z., Wei J., Shao D., Schwarz S., Wang S. (2013). Detection and new genetic environment of the pleuromutilin-lincosamide-streptogramin A resistance gene *lsa(E)* in methicillin-resistant *Staphylococcus aureus* of swine origin. J. Antimicrob. Chemother..

[45] Sarrou S., Liakopoulos A., Tsoumani K., Sagri E., Mathiopoulos K.D., Tzouvelekis L.S., Petinaki E. (2016). Characterization of a novel *lsa* (E)- and *lnu* (B)-carrying structure located in the chromosome of a *Staphylococcus aureus* sequence type 398 strain. Antimicrob. Agents Chemother..

[46] Yan X., Li Z., Chlebowicz M.A., Tao X., Ni M., Hu Y., Li Z., Grundmann H., Murray S., Pascoe B. (2016). Genetic features of livestock-associated *Staphylococcus aureus* ST9 isolates from Chinese pigs that carry the *lsa(E)* gene for quinupristin/dalfopristin resistance. Int. J. Med. Microbiol..

[47] Feßler A., Kadlec K., Wang Y., Zhang W.J., Wu C., Shen J., Schwarz S. (2018). Small antimicrobial resistance plasmids in livestock-associated methicillin-resistant *Staphylococcus aureus* CC398. Front. Microbiol..

[48] Sineke N., Asante J., Amoako D.G., Abia A.L.K., Perrett K., Bester L.A., Essack S.Y. (2021). *Staphylococcus aureus* in intensive pig production in South Africa: Antibiotic resistance, virulence determinants, and clonality. Pathogens.

[49] Pirolo M., Gioffrè A., Visaggio D., Gherardi M., Pavia G., Samel P., Ciambrone L., Di Natale R., Spatari G., Casalinuovo F. (2019). Prevalence, molecular epidemiology, and antimicrobial resistance of methicillin-resistant *Staphylococcus aureus* from swine in Southern Italy. BMC Microbiol..

[50] Ceballos S., Aspiroz C., Ruiz-Ripa L., Zarazaga M., Torres C. (2020). Antimicrobial resistance phenotypes and genotypes of methicillin-resistant *Staphylococcus aureus* CC398 isolates from Spanish hospitals. Int. J. Antimicrob. Agents.

[51] Kehrenberg C., Schwarz S. (2006). Distribution of florfenicol resistance genes *fexA* and *cfr* among chloramphenicol-resistant *Staphylococcus* isolates. Antimicrob. Agents Chemother..

[52] Monaco M., Pedroni P., Sanchini A., Bonomini A., Indelicato A., Pantosti A. (2013). Livestock-associated methicillin-resistant *Staphylococcus aureus* responsible for human colonization and infection in an area of Italy with high density of pig farming. BMC Infect. Dis..

[53] Larsen T.G., Samaniego Castruita J.A., Worning P., Westh H., Bartels M.D. (2024). Within-host genomic evolution of methicillin-resistant *Staphylococcus aureus* in long-term carriers. Appl. Microbiol. Biotechnol..

[54] Goyal M., Javerliat F., Palmieri M., Mirande C., van Wamel W., Tavakol M., Verkaik N.J., van Belkum A. (2019). Genomic evolution of *Staphylococcus aureus* during artificial and natural colonization of the human nose. Front. Microbiol..

[55] Hegstad K., Langsrud S., Lunestad B.T., Scheie A.A., Sunde M., Yazdankhah S.P. (2010). Does the wide use of quaternary ammonium compounds enhance the selection and spread of antimicrobial resistance and thus threaten our health?. Microb. Drug Resist..

[56] Luyckx K., Millet S., Van Weyenberg S., Herman L., Heyndrickx M., Dewulf J., De Reu K. (2016). A 10-day vacancy period after cleaning and disinfection has no effect on the bacterial load in pig nursery units. BMC Vet. Res..

[57] IBGE, Instituto Brasileiro de Geografia e Estatística (2017). Divisão Regional do Brasil em Regiões Geográficas Imediatas e Regiões Geográficas Intermediárias. Coordenação de Geografia.

[58] Clark A.E., Kaleta E.J., Arora A., Wolk D.M. (2013). Matrix-assisted laser desorption ionization-time of flight mass spectrometry: A fundamental shift in the routine practice of clinical microbiology. Clin. Microbiol. Rev..

[59] CLSI (2021). Performance Standards for Antimicrobial Susceptibility Testing.

[60] Zhang K., McClure J.A., Elsayed S., Louie T., Conly J.M. (2005). Novel multiplex pcr assay for characterization and concomitant subtyping of staphylococcal cassette chromosome *mec* types I to V in methicillin-resistant *Staphylococcus aureus*. J. Clin. Microbiol..

[61] Magiorakos A.P., Srinivasan A., Carey R.B., Carmeli Y., Falagas M.E., Giske C.G., Harbarth S., Hindler J.F., Kahlmeter G., Olsson-Liljequist B. (2012). Multidrug-resistant, extensively drug-resistant and pandrug-resistant bacteria: An international expert proposal for interim standard definitions for acquired resistance. Clin. Microbiol. Infect..

