# Supplementary material for: Multidrug-Resistant Staphylococcus aureus Colonizing Pigs and Farm Workers in Rio de Janeiro State, Brazil: Potential Interspecies Transmission of Livestock-Associated MRSA (LA-MRSA) ST398
Source: Antibiotics (Basel). 2024 Aug 14;13(8):767. doi: 10.3390/antibiotics13080767 (PMC11350785; doi:10.3390/antibiotics13080767)
Supplement: Supplementary file 1 [file antibiotics-13-00767-s001.zip › antibiotics-3071808-supplementary.pdf]

antibiotics

MDPI

Supplementary Materials

Table S1. Number of samples collected and of pigs and farm workers colonized with *S. aureus* in each farm investigated in the study.

Farm	Region	Collection date	Pig (n)		Farm work (n)	
			Investigated	Colonized	Investigated	Colonized
A	II	01/2014	10	2	2	0
B	II	01/2014	10	2	2	0
C	I	01/2014	10	0	1	0
D	II	01/2014	10	0	1	0
E	II	01/2014	10	2	-	-
F	III	01/2014	12	0	-	-
G[1]	I	01/2014	7	1	-	-
H	IV	01/2014	10	0	-	-
I	IV	01/2014	16	0	-	-
J	IV	01/2014	5	0	-	-
K	I	12/2015	21	1	5	2
L	III	11/2016	20	4	4	1
M	I	05/2019	10	1	1	0
N	III	06/2019	25	1	5	3
O[1]	I	08/2019	23	0	6	1
P	I	11/2019	31	0	4	0
Total			230	14	27*	7

[1]Farms A and B and farms G and O are localized in the same cities. * Four farms (A, B, C and D) were attended by the same veterinarian, and his sample was collected only once. Samples from animals and farm workers were collected on the same date on these four farms.

Table S2. Antimicrobial resistance patterns of multidrug-resistant *Staphylococcus aureus* strains from pigs and farm workers in pig farms from Rio de Janeiro state.

Region	Farm	Source	Host	Antimicrobial resistance pattern[2]
I	K	Pig	SN105	Chl (I), Cip, Cli, Ery, Nor, Pen, Sut (I), Tet
		Human	HSN10	iCli, Ery, Pen, Tet
	O	Human	HSN21	iCli, Ery, Gen, Pen
II	B	Pig	SN15	Chl, Cip, Cli, Ery, Pen, Tet
	E	Pig	SN51	Cef, Cip, Cli, Ery, Nor, Pen, Sut, Tet
		Pig	SN52	Cef, Cip, Cli, Ery, Nor, Pen, Tet[3]
III	L	Pig	SN128	Chl (I), Cip, Cli, Ery, Gen, Nor, Pen, Tet
		Pig	SN143	Chl (I), Cip, Cli, Ery, Gen, Nor, Pen, Sut, Tet
		Pig	SN144	Chl (I), Cip (I), Cli, Ery, Pen, Tet
		Pig	SN145[1]	**Chl, Cip, Cli, Ery, Gen, Nor, Pen, Tet**
				Chl, Cli, Ery, Pen, Sut, Tet
		Human	HSN12[1]	**Chl, Cip, Cli, Ery, Gen, Nor, Pen, Tet**
				Chl, Cip, Cli, Ery, Nor, Pen, Tet
				Chl (I), Cip, Cli, Ery, Nor, Pen, Tet
	N	Pig	SN182[1]	Chl,Cip, Cli, Tet
				**Chl,Cip, Cli, Ery, Pen, Tet**[4]
		Human	HSN16	Cip, Cli, Pen, Tet
			HSN18	**Chl,Cip, Cli, Ery, Pen, Tet**[4]

[1]Antimicrobial resistance pattern of more than one bacterial strain per pig or human; [2]Cef: cefoxitin (detection of MRSA, methicillin-resistant *Staphylococcus aureus*), Cip: ciprofloxacin, Cip (I): intermediate to ciprofloxacin, Chl: chloramphenicol; Chl (I): intermediate to chloramphenicol; Cli: clindamycin, iCli: inducible clindamycin resistance, Ery: erythromycin, Gen: gentamicin, Nor: norfloxacin, Pen: penicillin G, Sut: sulfamethoxazole-trimethoprim, Tet: tetracycline; [3]MRSA; [4]OS-MRSA (oxacillin-susceptible MRSA). Indistinguishable antimicrobial patterns of pig and human from the same farm are in bold.
